# Medical Schools and the American Medical Association

**Published:** 1849-11

**Authors:** 


### EDITORIAL DEPARTMENT.

*Medical Schools and the American Medical Association.*—In connection with some remarks under this caption, in a former number of the present volume of this Journal, we referred to some resolutions prepared by a member of the Association, from this city, Dr. James P. White, the publication of which was then deferred until the next number, for want of space. The resolutions were furnished by Dr. White, at our request, but were accidentally mislaid, and not recovered until a few days ago. This is our apology for not redeeming our promise until now.

The provisions contained in the resolutions, appear to us highly appropriate and judicious. They were submitted to several members of the Association, at the last meeting, and received their unqualified commendation. We hear frequently, from some quarters, sweeping assertions respecting the corrupt practices of medical schools. Now, if there be any foundation for such assertions, let the facts upon which they are based, be known. Justice to all parties demands this. To the medical profession it is due, in order that its patronage and influence shall receive a right direction. To the friends of medical education it is obviously due, whether in, or out of the medical profession. Exposure is certainly deserved by institutions to which such charges and insinuations will apply, if they are applicable to any, and it is eminently an act of justice to institutions meriting respect and consideration, that they should not be embraced in that indiscriminate censure and suspicion, which too often enters into declamations respecting medical reform, etc. The reasons for the resolutions, lucidly and succinctly set forth in the preamble, do not require amplification, and must commend themselves to the judgment of the candid reader.

We append a note by Dr. White, enclosing a copy of the resolutions:—

Dr. Flint,

*Dear Sir.*—The following preamble and resolutions were prepared with the intention of submitting them for consideration at the last meeting of the American Medical Association.

The unusual amount of miscellaneous business protracting the session much beyond the ordinary period, prevented me from submitting them at that time.

Being of opinion that something of the kind is demanded for the mutual protection of the profession, and the schools, and purposing to lay them before the Association at some subsequent meeting, I shall feel obliged to you for their insertion in the Journal, that the different members may bestow upon the subject such reflection as it deserves, and be prepared to make such suggestions as will conduce to the interests of all parties.

Yours truly,

JAMES P. WHITE.

*Whereas,* charges and insinuations are frequently made, imputing to medical institutions, venal conduct in selling degrees, or conferring them upon imperfect evidence of qualifications, and various other unworthy acts;

And *whereas,* such charges, if true, imply gross abuse of the corporate powers vested in collegiate institutions; are calculated to affect most seriously the cause of medical education, and are highly prejudicial to the character and interests of the medical profession;

And *whereas,* such charges and insinuations are usually applied to medical institutions, collectively, without discrimination or specifications, thus inflicting injustice, and bringing unmerited censure upon the colleges whose course is upright and honorable;

And *whereas,* malversations of medical schools are legitimate subjects for inquiry and investigation by the American Medical Association, with a view to such action as shall appear to be necessary for their correction, and at the same time to protect institutions deserving confidence and support;

And *whereas*, no committee at present exists to take cognizance of any abuses, although responsible individuals, actuated by praise-worthy motives, should desire to communicate knowledge of them;

Therefore *resolved*, that a committee of seven members of the Association, not connected with the medical schools, be appointed by the president, to receive any specific charges of direliction of duty, and propriety, by medical institutions; and whose duty it shall be to investigate these charges, and to report all the important facts and circumstances therewith connected; and such action as they shall recommend, at the next meeting of this Association.

*Resolved*, that it is hereby enjoined as a duty incumbent on every member of the Association, to communicate to this committee any information having important bearing upon the objects for which the committee is created. And any member of this committee is fully authorized to call upon any member of the Association, for any such information in his possession.

*Resolved*, that it shall be considered improper, and a violation of medical ethics, for members of the Association, to make or repeat charges against the character of chartered medical institutions, while they do not give them specific form, and report them for the action of the committee hereby constituted.

